# Landiolol, an intravenous β1‐selective blocker, is useful for dissociating a fusion of atrial activation via accessory pathway and atrioventricular node

**DOI:** 10.1002/joa3.12934

**Published:** 2023-10-03

**Authors:** Takahiko Kinjo, Masaomi Kimura, Noriyoshi Kaname, Daisuke Horiuchi, Taihei Itoh, Yuji Ishida, Kimitaka Nishizaki, Yuichi Toyama, Shingo Sasaki, Hirofumi Tomita

**Affiliations:** ^1^ Department of Cardiology and Nephrology Hirosaki University Graduate School of Medicine Hirosaki Japan; ^2^ Department of Advanced Management of Cardiac Arrhythmias Hirosaki University Graduate School of Medicine Hirosaki Japan; ^3^ Department of Cardiac Remote Management System Hirosaki University Graduate School of Medicine Hirosaki Japan; ^4^ Department of the Advanced Therapeutics for Cardiovascular Diseases Hirosaki University Graduate School of Medicine Hirosaki Japan

**Keywords:** accessory pathway, atrioventricular node, beta‐blocker, fusion of atrial activation

## Abstract

**Introduction:**

During ventricular pacing, a fusion of atrial activation may occur owing to the simultaneous retrograde conduction of the atrioventricular (AV) node and accessory pathway (AP), potentially leading to an inaccurate mapping of the atrial AP insertion site.

**Objective:**

We tested the hypothesis that landiolol, an ultra‐short‐acting intravenous β1‐blocker, could dissociate a fusion of atrial activation.

**Methods:**

We conducted a prospective before‐and‐after study to investigate the effect of landiolol on retrograde conduction via the AV node and AP. We enrolled 21 consecutive patients with orthodromic AV reciprocating tachycardia who underwent electrophysiological studies at our hospital between January 1, 2018, and August 31, 2020.

**Results:**

Six patients exhibited a fusion of atrial activation. After landiolol administration (10 μg/kg/min), the effective refractory period was unchanged in AP (280 [240–290] ms vs. 280 [245–295] ms, *p* = .91), whereas that of the AV node was prolonged (275 [215–380] ms vs. 332 [278–445] ms, *p* = .03). The Wenckebach pacing rate via retrograde AV node decreased after landiolol administration (180 [140–200] beats per minute [bpm] vs. 140 [120–180] bpm, *p* = .02). Thus, landiolol decreased the minimum ventricular pacing rate required to dissociate a fusion of atrial activation (180 [160–200] bpm vs. 140 [128–155] bpm, *p* = .007). Radiofrequency catheter ablation under landiolol administration successfully eliminated AP in all patients during ventricular pacing without complications or recurrence.

**Conclusion:**

Landiolol inhibited the AV node without affecting the AP and helped dissociate a fusion of atrial activation at a lower ventricular pacing rate.

## Graphical abstract

During ventricular pacing, a fusion of atrial activation may occur owing to the simultaneous retrograde conduction of the atrioventricular (AV) node and accessory pathway (AP), potentially leading to an inaccurate mapping of the atrial AP insertion site (A). Landiolol inhibited the AV node without affecting the AP and helped dissociate a fusion of atrial activation (B) at a lower ventricular pacing rate.

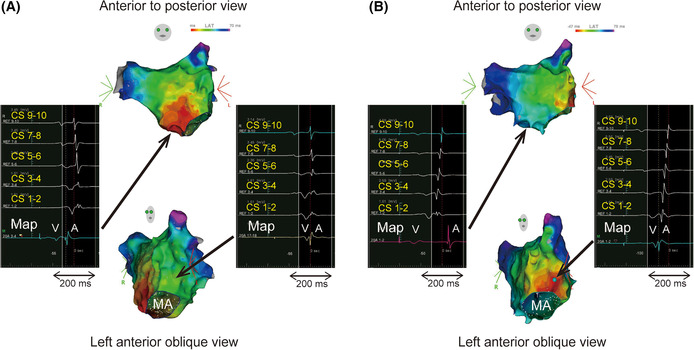

## 1 INTRODUCTION

Radiofrequency catheter ablation of the atrioventricular (AV) accessory pathway (AP) is an established therapy. Several methods have been reported^1^, ^2^, ^3^, ^4^; however, the atrial insertion site is sometimes the only target when the AP only exhibits retrograde conduction. Mapping of the AP atrial insertion site is widely performed during ventricular pacing; however, the fusion of atrial activation caused by simultaneous retrograde conduction of the AV node and AP has been reported.^5^, ^6^, ^7^ A fusion of atrial activation can lead to misleading the mapping results; however, it can be dissociated by prolonging retrograde conduction via the AV node by ventricular extrastimulus,^6^ overdrive pacing at a high rate,^7^ or the pharmacologic block.^5^ These approaches were discovered before catheter ablation became widely accepted. Therefore, the suitable methods for dissociating a fusion of atrial activation in the catheter ablation era remain to be elucidated.

Landiolol is an intravenous β1‐receptor‐selective blocker with a short half‐life (3.5 min) that is available in Japan^8^ and Europe.^9^ We hypothesized that landiolol might have a potential role in mapping AP when a fusion of atrial activation is observed because of its short half‐life. Thus, we conducted a prospective study to test the hypothesis that landiolol can dissociate a fusion of atrial activation by inhibiting the AV node without affecting the AP. We aimed (1) to investigate the effect of landiolol on the minimum ventricular pacing rate required to dissociate a fusion of atrial activation and the effective refractory period (ERP) or conduction times of the AP and AV node, and (2) to evaluate the efficacy and safety of landiolol in catheter ablation of the AP.

## 2 METHODS

### 2.1 Study design

This was a prospective, single‐center, quasi‐experimental, nonrandomized before‐and‐after study. The study intervention was intravenous landiolol administration during the electrophysiological study and catheter ablation. We enrolled patients aged 15–80 years admitted to Hirosaki University Hospital between January 1, 2018, and August 31, 2020, for catheter ablation of paroxysmal supraventricular tachycardia (PSVT). After the electrophysiological study, patients with orthodromic AV reciprocating tachycardia (ORT) involving an AP were included. We excluded patients: (1) with an allergy to landiolol, (2) with contraindications for β‐blocker (such as uncontrolled bronchial asthma, shock, coronary spastic angina, and peripheral artery disease), (3) with a baseline heart rate of 40 beats/min or less at baseline, (4) with left ventricular ejection fraction 25% or less, and (5) who were unwilling to participate in the study or were deemed inappropriate for other reasons by the physician. Patients with decremental or multiple AP and intermittent preexcitation syndrome were also excluded.

### 2.2 Electrophysiological study and definition of a fusion of atrial activation

The study was conducted with participants in a fasting and unsedated state. All antiarrhythmic drugs were discontinued for at least five half‐lives before the procedure. Quadripolar or decapolar electrode catheters were positioned in the right ventricular (RV) apex and His‐bundle (HB) region using the femoral venous access. A duodecapolar catheter was inserted via the internal jugular vein and placed into the coronary sinus (CS), and recorded the electrograms of the CS and high right atrium.

Tachycardia was induced by programmed atrial or ventricular stimulation using a cardiac stimulator (BC‐1100; Fukuda Denshi Co., Ltd.). ORT diagnoses were based on previously reported electrophysiological criteria^10^, ^11^, ^12^ and catheter ablation results. A fusion of atrial activation was defined as a difference in the atrial activation sequence between during tachycardia and ventricular pacing, resulting from simultaneous retrograde conduction of both the AV node and the AP.

### 2.3 Intravenous infusion of landiolol and catheter ablation

The ERP and conduction time of the retrograde AP were measured. The ventriculoatrial (VA) interval, representing the conduction time of the retrograde AV node or AP, was defined as the duration from the onset of the ventricular electrogram in the RV catheter to the earliest atrial activation site in the positioned catheters. In the patients with a fusion of atrial activation, the minimum ventricular pacing rate required to dissociate the fusion of atrial activation was also evaluated.

After obtaining the baseline data, landiolol (10 μg/kg/min) was administered, and data were collected repeatedly. In the previous study from healthy male volunteers,^8^ the 10 μg/kg/min dose did not cause a change in resting heart rate, there were no adverse events, and we were concerned about the insufficiency of the effect of landiolol in lower doses. Thus, we set the dose to the maximum dose (10 μg/kg/min) from the clinical use in the cardiac care unit in Japan.^13^ Radiofrequency catheter ablation of the AP was performed during landiolol administration using a three‐dimensional (3D) electroanatomic mapping system (CARTO 3, Biosense Webster, Johnson & Johnson). Subsequently, the ERP and VA interval for retrograde conduction via the AV node was measured during landiolol administration (10 μg/kg/min) and after the discontinuation. All data were measured more than 20 min after starting or discontinuing landiolol administration, which was considered to be in a steady state.^8^ Following catheter ablation and data measurement, intravenous isoproterenol (0.5–1.0 μg/min) was continuously administered, and a waiting period of at least 30 min was observed to verify the absence of conduction via the AP.

### 2.4 Follow‐up

Follow‐up data for all patients, such as symptom recurrence, 12‐lead electrocardiography, and morbidity/mortality, were obtained from our outpatient clinic.

### 2.5 Statistical analysis

The minimum required sample size was determined using JMP Pro version 14 (SAS Institute Inc). We considered a change of 30 ms in the ERP, conduction time, or minimum ventricular pacing rate required to dissociate a fusion of atrial activation after administering landiolol (10 μg/kg/min) as clinically significant. Assuming a standard deviation of the mean difference of 20 ms, we calculated that a sample size of at least 17 patients with ORT would be necessary to achieve 80% statistical power and an *α* error of <5%.

Continuous variables were expressed as the median (interquartile range), and categorical variables were expressed as the number and percentage of patients. Data comparisons between the patients with and without a fusion of atrial activation were performed using the Mann–Whitney *U* or Fisher's exact test. Comparisons of ERP, conduction time, and minimum ventricular pacing rate dissociating a fusion of atrial activation before and after landiolol (10 μg/kg/min) administration were performed using the Wilcoxon matched‐pairs signed‐rank test. A repeated‐measures two‐way analysis of variance was conducted to examine the effect of pacing rate and landiolol on the AP conduction time. Receiver operator characteristic (ROC) curves were used to determine the threshold of AH interval or tachycardia cycle length to predict a fusion of atrial activation. The ROC cutoff value was calculated by Youden Index. The data were analyzed with GraphPad Prism version 9.2.0 (GraphPad Software). Statistical significance was set at *p* < .05.

### 2.6 Ethics

Written informed consent was obtained from all patients before the electrophysiological study. This study was approved by the Committee of Medical Ethics of Hirosaki University Graduate School of Medicine, Hirosaki, Japan (Aomori, Japan; reference number: 02H‐29006) and was conducted in accordance with the Helsinki Declaration. The trial was registered with the University Hospital Medical Information Network (registration number: UMIN000030753).

## 3 RESULTS

### 3.1 Patients' characteristics

**FIGURE 1:**
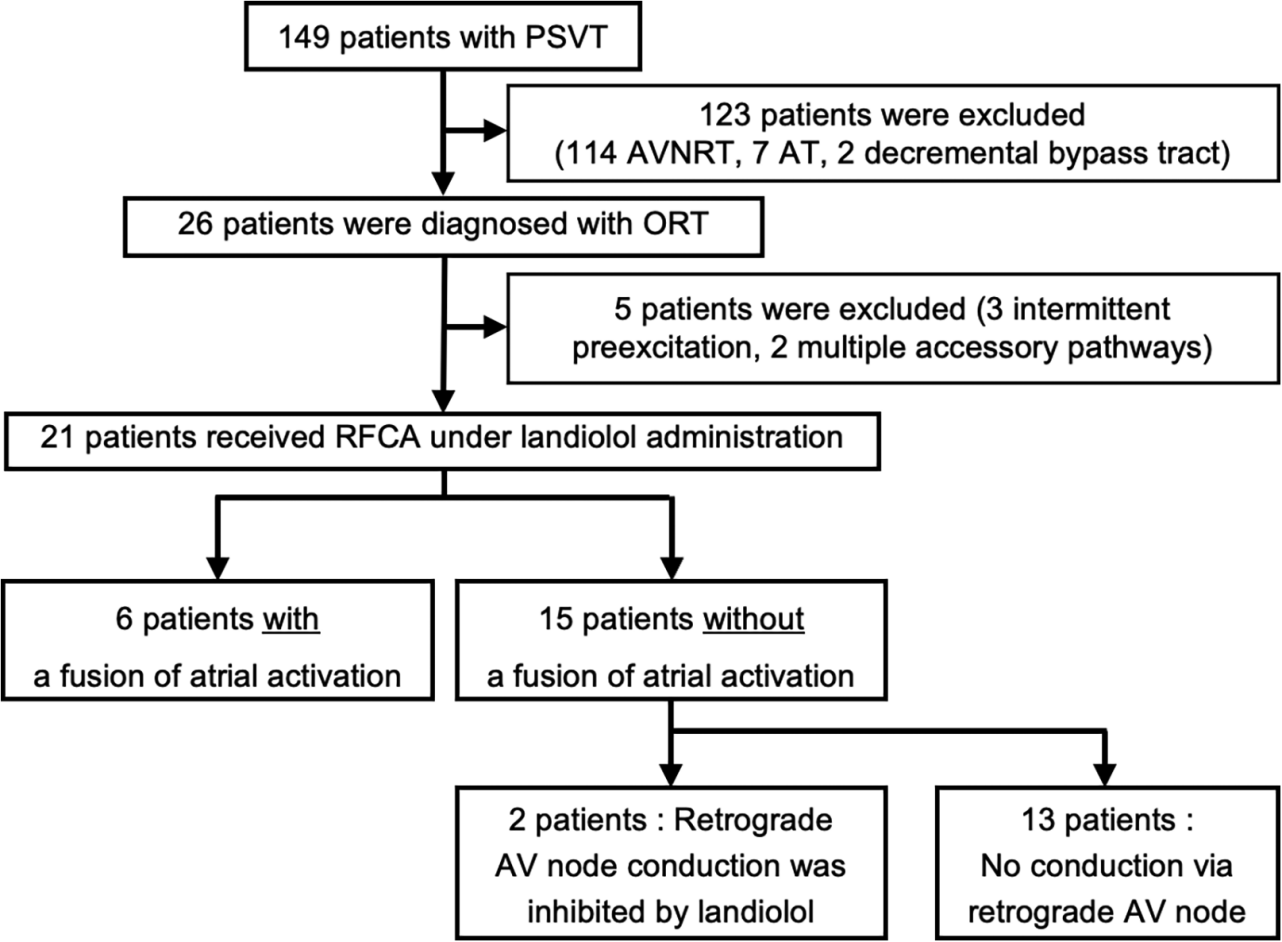
Study flow diagram. AT, atrial tachycardia; AV, atrioventricular; AVNRT, atrioventricular nodal reentrant tachycardia; ORT, orthodromic atrioventricular reciprocating tachycardia; PSVT, paroxysmal supraventricular tachycardia; RFCA, radiofrequency catheter ablation.

Figure 1 depicts a flowchart on the inclusion and exclusion of participants in this study. We enrolled 149 patients with PSVT during the study period. Of these, 123 were excluded from this study because 114 patients were diagnosed with atrioventricular nodal reentrant tachycardia, seven with atrial tachycardia, and two with decremental bypass tract. Twenty‐six patients were diagnosed with orthodromic AV reciprocating tachycardia (ORT) using a retrograde accessory pathway. Of them, three patients with intermittent preexcitation syndrome and two patients with multiple accessory pathways were excluded.

Finally, this study included 21 patients with ORT who received landiolol administration. Retrograde AV node conduction was detected in six patients with a fusion of atrial activation. The remaining 15 patients did not exhibit a fusion of atrial activation, two had retrograde AV node conduction in the control condition but were completely inhibited by landiolol, and 13 had no retrograde AV node conduction in the control condition and under landiolol administration.

Table 1 presents the patients' characteristics. Patients with a fusion of atrial activation had a shorter baseline AH interval and tachycardia cycle length (Table 1). In the ROC curves for prediction of a fusion of atrial activation, the cutoff point of AH interval and tachycardia cycle length was 63 ms (area under the curve [AUC]: 0.95, 95% confidence interval [CI]: 0.84 to 1.0) and 316 ms (AUC 0.87, 95% CI: 0.67 to 1.0), respectively (Figure S1).

**TABLE 1:** Baseline characteristics of study patients.

	Atrial activation fusion (+) *n* = 6	Atrial activation fusion (−) *n* = 15	*p*‐value
Age	55 (42–57)	54 (45–62.5)	.48
Male (%)	5 (83%)	6 (46%)	.18
Body mass index	23.5 (18.3–28.4)	23.4 (21.3–25.4)	.79
Echocardiography data			
Left ventricular ejection fraction (%)	65 (62–69)	66 (62–69)	.96
Left atrium diameter (mm)	32.5 (28.5–35.8)	32.0 (31.0–37.6)	.82
Comorbidity			
Structure heart disease	0	0	
Paroxysmal atrial fibrillation (%)	2 (33%)	1 (8%)	
Others	1 (arteriosclerosis obliterans)	2 (1; Graves' disease, 1; sick sinus syndrome)	
Baseline electrophysiological data			
AH (ms)	60 (48–67)	83 (74–89)	.002
HV (ms)	42 (41–49)	42 (36–53)	.42
VA (during RV pace 100 ppm)	154 (121–165)	160 (112–172)	.75
Tachycardia cycle lengths (ms)	295 (271–345)	375 (327–410)	.02
AH (during tachycardia) (ms)	132 (99–188)	203 (175–265)	.07
VA (during tachycardia) (ms)	50 (50–57)	82 (55–105)	.03

*Note*: Data are presented as median (interquartile range) or *n* (%).

Abbreviations: AH, atrial‐His; HV, His‐ventricular; RV, right ventricle; VA, ventricular atrial.

### 3.2 Features of a fusion of atrial activation and its dissociation

**FIGURE 2:**
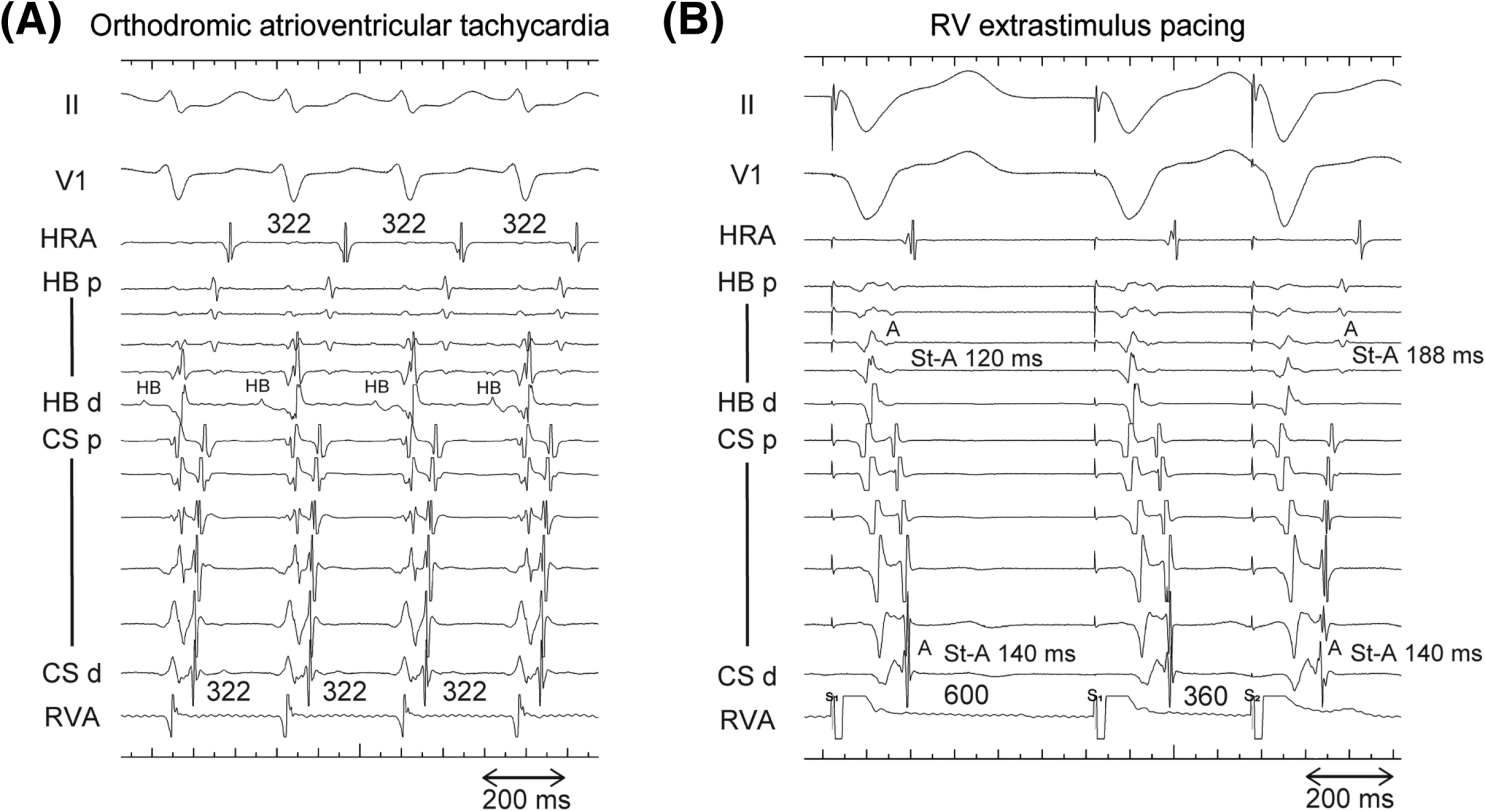
Example of a fusion of atrial activation in a patient with a left lateral accessory pathway. (A) Intracardiac electrogram during ORT using the left lateral accessory pathway. The accessory pathway fully activates the atrium. (B) Intracardiac electrogram during right ventricular (RV) extrastimulus pacing. Under the basic cycle lengths (600 ms), the His‐bundle (HB) catheter and distal coronary sinus (CS) exhibited short ventriculoatrial (VA) intervals, indicating that both the AV node and accessory pathway activated the atrium. After the extrastimulus with a coupling interval of 360 ms, only the accessory pathway activated the atrium because the atrial activation sequence was the same as that during tachycardia. CS, coronary sinus; HB, His‐bundle; HRA; high right atrium; RV, right ventricle; RVA, right ventricular apex; VA, ventriculoatrial; other abbreviations are in Figure 1.

**FIGURE 3:**
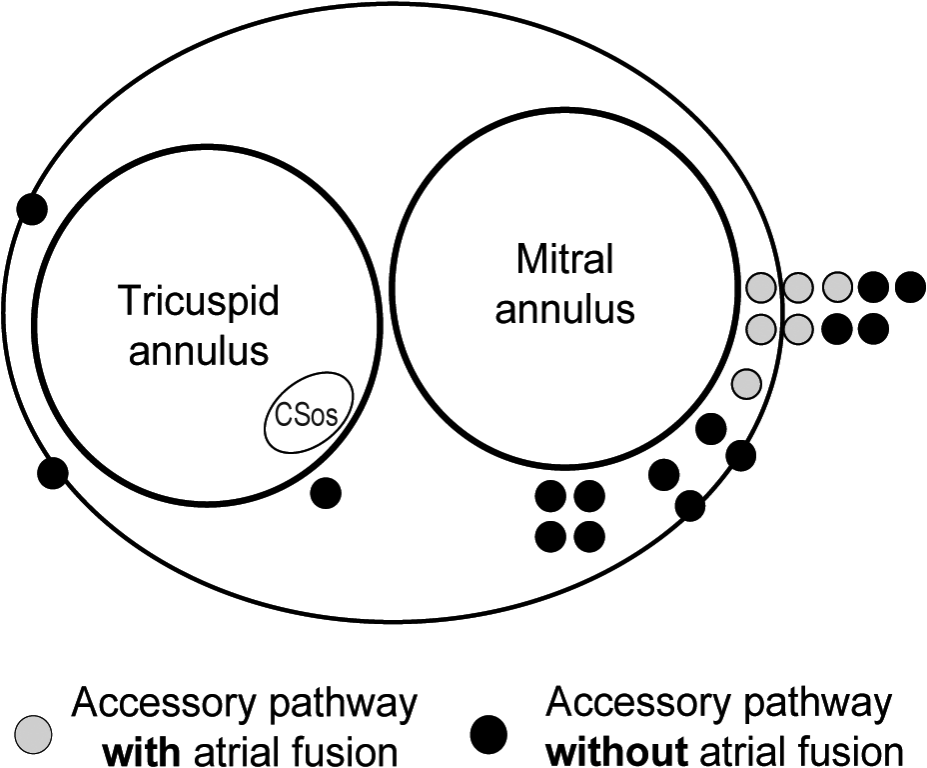
Distribution of accessory pathway with and without a fusion of atrial activation. The black dots indicate the accessory pathways that do not exhibit a fusion of atrial activation. The gray dots indicate the accessory pathways that exhibit a fusion of atrial activation. CSos, coronary sinus ostium.

Figure 2 illustrates an example of a fusion of atrial activation. Retrograde conduction solely occurred through the left lateral AP during ORT (Figure 2A). When RV pacing was conducted at the basic cycle length (600 ms), the HB catheter and distal CS exhibited short VA intervals, indicating that the AV node and AP activated the atrium. After the extrastimulus with a coupling interval of 360 ms, only the AP activated the atrium, as evidenced by matching the atrial activation sequence observed during tachycardia (Figure 2B). Figure 3 illustrates the distribution of the AP locations among the study patients. A fusion of atrial activation was observed only in patients with a left lateral or posterolateral AP.

**FIGURE 4:**
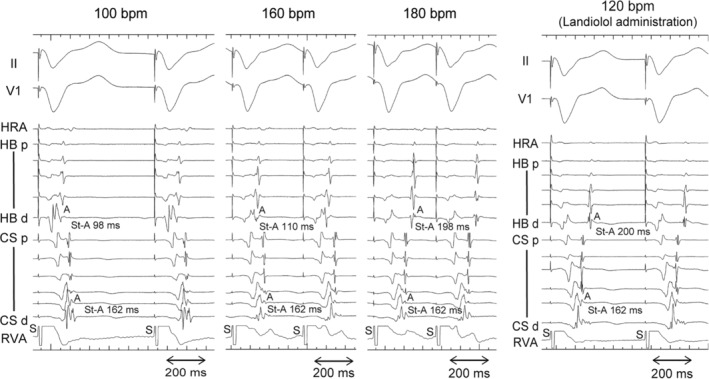
An example of the dissociation of a fusion of atrial activation. During the RV pacing at 100 beats per minute (bpm), the earliest atrial activation site was the HB catheter. As the pacing rate increased to 160 bpm, the VA interval recorded at the HB catheter was prolonged, whereas that of the distal CS remained unchanged. RV pacing at 180 bpm; the atrial activation sequence of the CS changed from distal to proximal, indicating the accessory pathway conduction. After the intravenous administration of landiolol, dissociating a fusion of atrial activation was achieved at 120 bpm. bpm, beats per minute; other abbreviations are in Figures 1 and 2.

**FIGURE 5:**
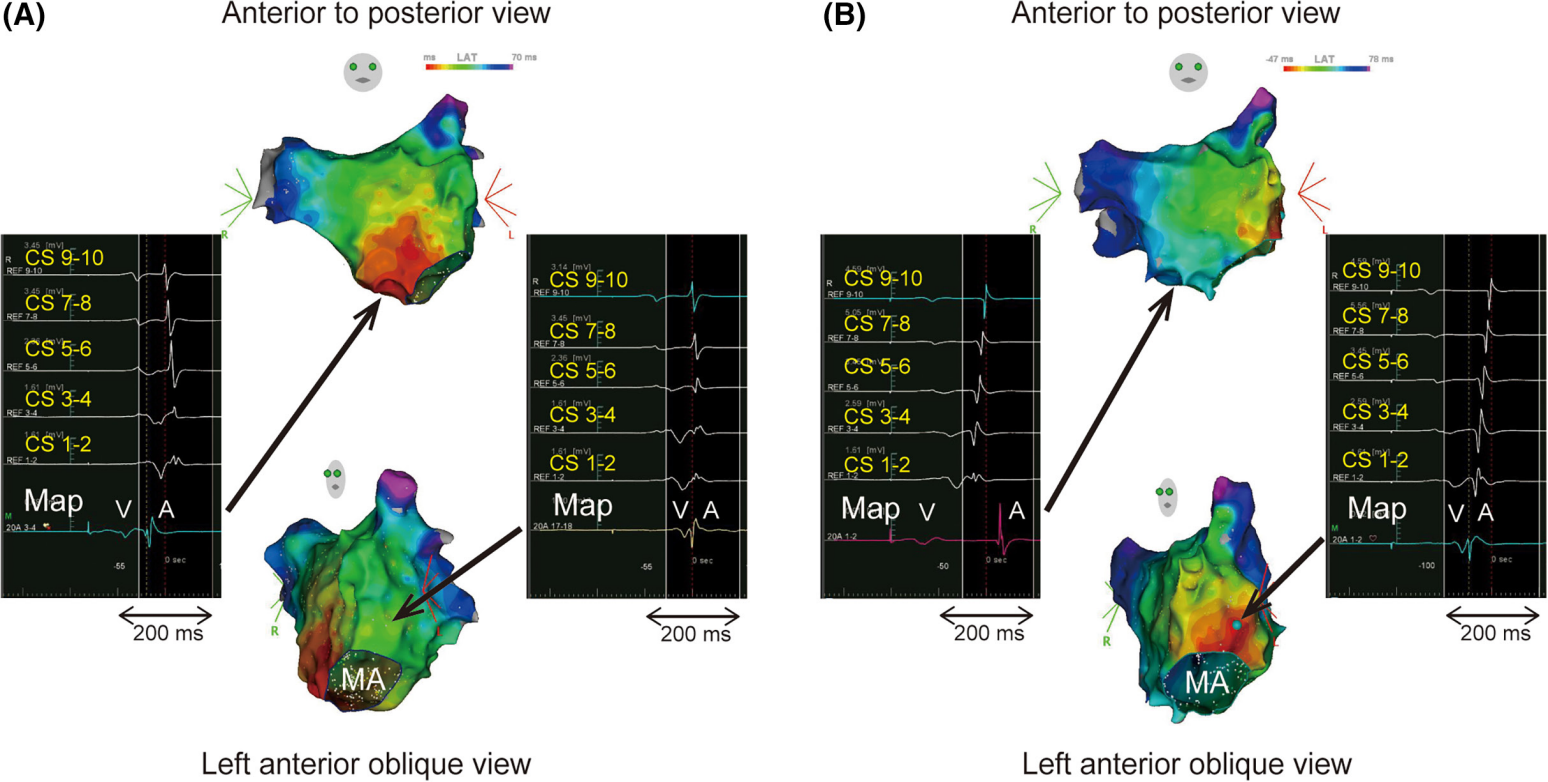
Three‐dimensional activation map of the left atrium in a patient with a fusion of atrial activation involving the left lateral accessory pathway. (A) Left atrial activation map during RV pacing at 100 bpm. The proximal‐to‐distal atrial sequence at the CS indicates that the conduction via the AV node is dominant. The VA interval in the left lateral mitral annulus is short; however, the atrial septum is the earliest activation site. (B) Left atrial activation map during RV pacing at 120 bpm under landiolol (10 μg/kg/min) administration. The distal‐to‐proximal atrial sequence at the CS excludes the fusion of atrial activation. The earliest atrial activation site is the left lateral mitral annulus. MA, mitral annulus; other abbreviations are in Figures 1, 2, and 4.

Figure 4 depicts a representative example of the dissociation of a fusion of atrial activation involving the left lateral AP. During RV pacing at 100 beats per minute (bpm), the atrial activation sequence differed from that observed during ORT, indicating concurrent atrium activation by the AV node and AP. In this condition, the earliest atrial activation site was the atrial septum, suggesting retrograde conduction via the AV node, even when employing a 3D mapping system (Figure 5A). As the pacing rate increased, the VA interval recorded at the HB catheter was prolonged, whereas that of distal CS remained unchanged (Figure 4). The minimum ventricular pacing rate required to dissociate a fusion of atrial activation was 180 bpm; however, after intravenous administration of landiolol, this threshold was achieved at 120 bpm (Figure 4). In this condition, the 3D mapping system revealed that the atrial insertion site of the AP was the earliest atrial activation site (Figure 5B).

### 3.3 Effect of landiolol in the retrograde conduction via AP and AV node

**FIGURE. 6:**
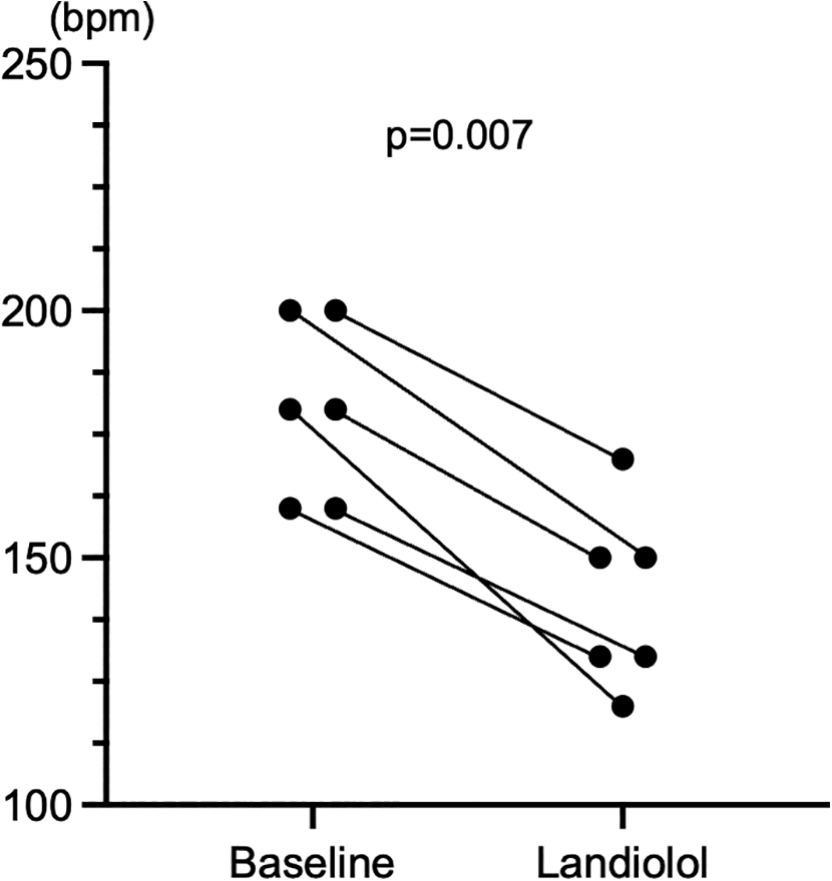
Minimal ventricular pacing rate requiring dissociation of a fusion of atrial activation. At baseline, the minimal ventricular pacing rate required to dissociate a fusion of atrial activation was high, which significantly decreased after landiolol administration. bpm, beats per minute.

**FIGURE 7:**
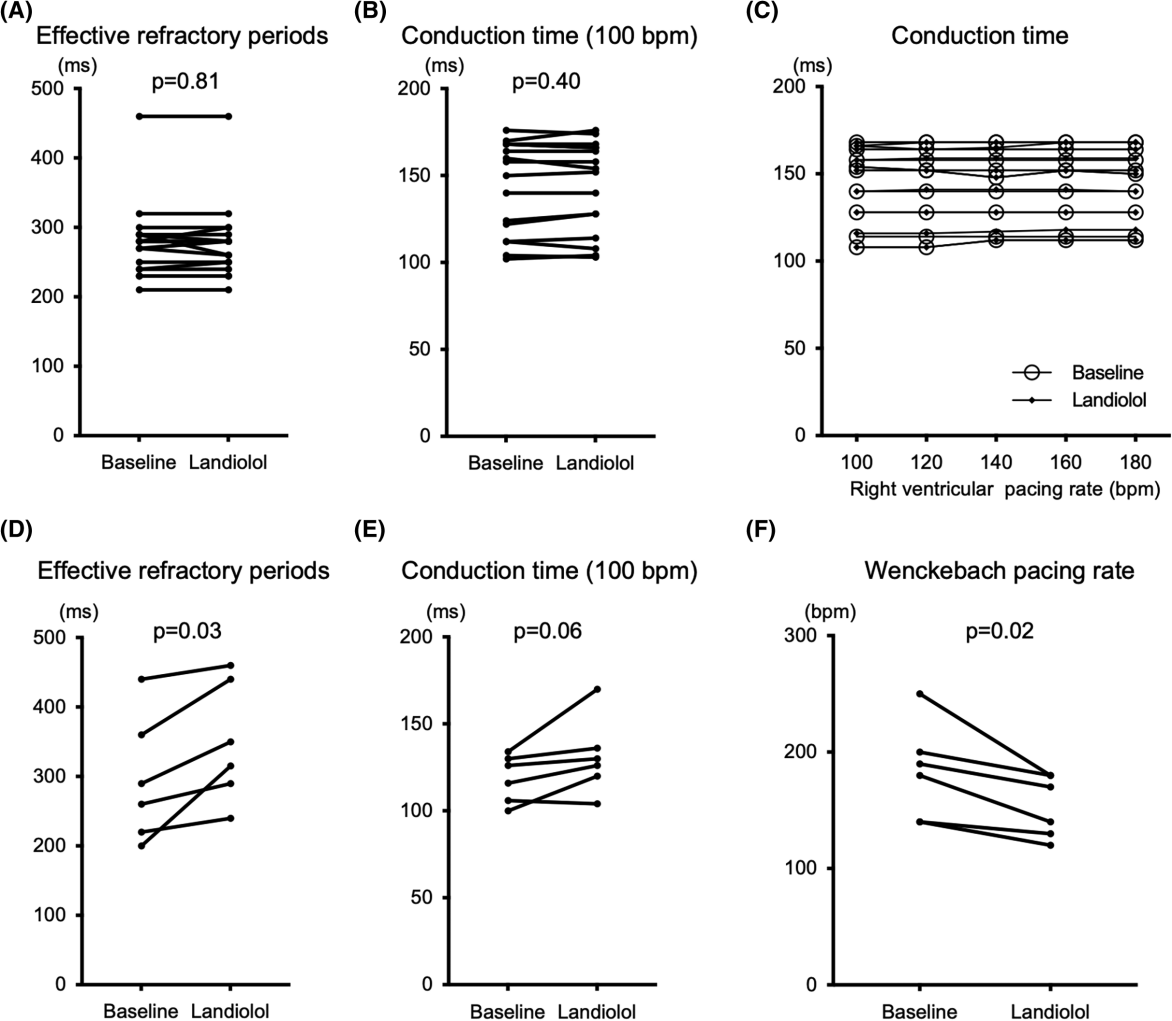
Effect of landiolol on the retrograde conduction via accessory pathway (A–C) and AV node (D–F). (A) The effective refractory period (ms) of the conduction via the retrograde accessory pathway was unchanged after landiolol administration. (B) Conduction time (ms) via the retrograde accessory pathway was unchanged after landiolol administration. (C) Conduction time (ms) via the retrograde accessory pathway remained unchanged after landiolol administration, even if the RV pacing rate increased. No significant differences were observed in the RV pacing rate factor, the landiolol factor, and the interaction effects. (D) The effective refractory period (ms) of the conduction via the retrograde AV node was prolonged after landiolol administration. (E) Conduction time (ms) via the retrograde AV node was prolonged; however, it was insignificant after landiolol administration. (F) The Wenckebach pacing rate (bpm) via retrograde AV nodal conduction decreased after landiolol administration. All abbreviations are in Figures 1, 2, and 4.

The minimal ventricular pacing rate dissociating a fusion of atrial activation was high at baseline; however, landiolol decreased the minimum ventricular pacing rate (180 [160–200] vs. 140 [128–155], *p* = .007) (Figure 6). Figure 7 presents the results regarding the ERP and conduction time of retrograde AP and AV node, and the Wenckebach pacing rates of the retrograde AV node. Before and after landiolol administration, the ERP (280 [240–290] ms vs. 280 [245–295] ms, *p* = .91) and conduction time (158 [117–168] ms vs. 154 [121–167] ms, *p* = .67) of AP were unchanged (Figure 7A,B). The conduction time via AP was unchanged even though the pacing rate increased before and after landiolol administration (Figure 7C). The ERP of the AV node was prolonged after landiolol administration (275 [215–380] ms vs. 332 [278–445] ms, *p* = .03) (Figure 7D). The conduction time of the AV node was prolonged but insignificant after landiolol administration (121 [105–131] ms vs. 128 [116–146] ms, *p* = .06) (Figure 7E). The Wenckebach pacing rate of retrograde AV node conduction decreased after landiolol administration (180 [140–200] bpm vs. 140 [120–180] bpm, *p* = .02) (Figure 7F).

### 3.4 The outcome of catheter ablation and follow‐up

All the patients underwent radiofrequency catheter ablation during RV pacing under continuous intravenous infusion of landiolol. The current was initiated at 20 W and increased to 30 W. Acute success was achieved in all the patients. No adverse events, including landiolol‐related adverse events, were observed. During the mean follow‐up period of 51 ± 24 weeks, no patient experienced tachycardia recurrence.

## 4 DISCUSSION

### 4.1 Major findings

We prospectively examined the role of landiolol in electrophysiological study and catheter ablation of AP with a fusion of atrial activation. Several findings of this study are novel contributions to the literature. First, a fusion of atrial activation was observed in 4% of patients with PSVT and 23% of patients with ORT. Second, landiolol decreased the minimum ventricular pacing rate required to dissociate a fusion of atrial activation by prolonging the ERP or decreasing the Wenckebach pacing rate of the retrograde AV node conduction without affecting conduction via the AP. Third, radiofrequency catheter ablation was successful under landiolol administration without complications.

### 4.2 Possible mechanism of a fusion of atrial activation

A fusion of atrial activation was caused by the good conductivity of the retrograde AV node. The baseline AH interval was shorter in the patients with a fusion of atrial activation, suggesting better conductivity of the AV node in these patients. Retrograde AV node conduction persisted after landiolol administration in patients with a fusion of atrial activation. In contrast, retrograde AV node conduction was either absent or completely inhibited following landiolol administration in patients without a fusion of atrial activation. Additionally, patients with a fusion of atrial activation exhibit a shorter tachycardia cycle length, which could be beneficial for preprocedural prediction. No differences were observed in age, gender, echocardiographic data, or the prevalence of structural or valvular heart disease between groups with and without a fusion of atrial activation.

Due to the predominance of left‐side AP among participants in this study and the limited presence of septal or right‐side AP, it was not feasible to investigate the potential correlation between AP location and atrial activation fusion despite its relevance is noteworthy.

### 4.3 Pharmacologic effect of landiolol on AV node and AP

Our study revealed that landiolol inhibits retrograde AV nodes without affecting AP conduction. The ERP and conduction time of the AP were unchanged after landiolol administration in all patients (Figure 7D–F). Landiolol inhibited the retrograde AV node conduction in two patients. Among the six cases with retrograde AV node conduction, the ERP of the AV node was prolonged, and the Wenckebach pacing rate of the AV node decreased (Figure 7D,F). Therefore, landiolol decreased the minimum ventricular pacing rate required to dissociate a fusion of atrial activation. The conduction time (during RV pacing at 100 bpm) of the retrograde AV node was prolonged but insignificant. Landiolol might enhance the prolongation of conduction via retrograde AV node as the pacing rate increases. However, statistical analysis was not feasible because of limited data on higher pacing rates due to the Wenckebach block.

### 4.4 Comparison with previous studies

Eleven percent of patients with antegrade AP exhibit unapparent preexcitation syndrome, characterized by no preexcitation at sinus rhythm that manifests during atrial pacing.^14^ This phenomenon occurs when anterograde conduction via the AV node conceals conduction via the AP. It was prevalent in patients with a left lateral AP located farther from the sinus node. The fusion of atrial activation observed in our study exhibited a phenomenon similar to the previous report at the atrium during RV pacing. Furthermore, another study has demonstrated that excluding ventricular activation fusion caused by conduction via the AP and AV node by atrial overdrive pacing enables a more accurate prediction of AP localization using 12‐lead electrocardiography.^15^ Similarly, our study demonstrated the importance of excluding a fusion of atrial activation when mapping the atrial insertion site of the AP.

Several drugs have been reported to be AV node blockers that do not inhibit AP conduction. Adenosine has been reported to manifest anterograde AP conduction in intermittent or unapparent preexcitation syndrome, as described in several small case series.^16^, ^17^ However, an intravenous bolus of adenosine may be inappropriate for AP mapping because of its short half‐life. Similar to our study, propranolol (0.1 mg/kg), an intravenous nonselective β‐blocker, has been reported not to affect the ERP of AP.^18^, ^19^ Conversely, several investigators have reported that isoproterenol infusion shortened the ERP of AP.^20^ Based on the results of these reports, we postulated that AP is sensitive to catecholamine but to lesser extent than the AV node. In this context, a higher dosage of β‐blockers might affect the ERP or conduction time of AP. Therefore, we deemed landiolol, with its ability to adjust the dosage owing to its short half‐life, more suitable for invasive electrophysiological study and catheter ablation.

### 4.5 Clinical implications

Landiolol may have a role in radiofrequency catheter ablation procedures targeting the AP by lowering the ventricular pacing rate required for dissociation in patients with a fusion of atrial activation. Following an extensive literature review, this is the first study of radiofrequency catheter ablation of the AP performed during landiolol administration. This approach proved to be safe, with no complications or instances of late recurrence. One of the most common causes of failed AP ablation is catheter instability.^21^ Radiofrequency energy delivery during ORT is not recommended owing to potential catheter dislodgement caused by sudden tachycardia termination. Similarly, extrastimulus may be unsuitable for catheter instability. A high rate of constant ventricular or entrainment pacing^22^ may alleviate this concern; however, an excessively high pacing rate is required to dissociate a fusion of atrial activation and may pose difficulties to the patients.

Additionally, based on the study results, it would be better to be aware of this phenomenon in cases with short tachycardia cycle length or left‐sided AP.

### 4.6 Study limitations

This study had some limitations. First, although we introduced a washout period, the carryover effects of landiolol may have influenced the results of our study. A more appropriate study design would have been a crossover design; however, we did not apply it owing to concerns about doubling the study duration. Second, we could not investigate the dose–response of conduction via AP and AVN to landiolol owing to concerns about prolonging the study duration. Third, the sympathetic tone may have weakened landiolol effects because this study was performed in an unsedated state. Fourth, changing the pacing site to the basal RV or left ventricle may have affected the incidence of a fusion of atrial activation. Fifth, more patients than expected had no retrograde AV node conduction at baseline or under landiolol administration resulting in a smaller sample size available for statistical analysis. Sixth, we were unable to examine the risk factor of a fusion of atrial activation because of the small sample size. Seventh, because our patient population includes more AVNRT patients and fewer ORT patients than previously reported,^11^ cautions should be taken in assessing the proportion of patients presenting with a fusion of atrial activation. However, despite these limitations, the purpose of this study was achieved because it demonstrated that landiolol decreased the minimum ventricular pacing rate dissociating a fusion of atrial activation.

## 5 CONCLUSIONS

Landiolol, a β1‐selective blocker, inhibited the AV node without affecting the AP and helped dissociate a fusion of atrial activation at a lower ventricular pacing rate. Therefore, landiolol may have a role in radiofrequency catheter ablation procedures targeting the AP by lowering the ventricular pacing rate required for dissociation in this patient population.

## AUTHOR CONTRIBUTIONS

Takahiko Kinjo conceived the idea of the study, developed the statistical analysis plan, conducted statistical analyses, and drafted the original manuscript. Noriyoshi Kaname advised on research ideas. Masaomi Kimura supervised the conduct of this study. All authors contributed to the interpretation and analysis of the results, reviewed and revised the manuscript draft, and approved the final version for submission.

## FUNDING INFORMATION

This research received no specific grant from funding agencies in the public, commercial, or not‐for‐profit sectors.

## CONFLICT OF INTEREST STATEMENT

Dr. Masaomi Kimura is an associate professor, and Dr. Taihei Itoh is an associate professor/lecturer of the Department of Advanced Management of Cardiac Arrhythmias, which is an endowment department supported by Medtronic Japan Co., Ltd., Japan Lifeline Co., Ltd, and Fukuda Denshi Kita‐Tohoku Hanbai Co., Ltd. Dr. Yuji Ishida is an assistant professor of the Department of Cardiac Remote Management System, which is an endowment department supported by BIOTRONIK Japan Co., Ltd. Dr. Shingo Sasaki received research grant from Boston Scientific Japan Co., Ltd. and is a concurrent associate professor of the Department of Advanced Management of Cardiac Arrhythmias and the Department of Cardiac Remote Management System. Dr. Hirofumi Tomita is a concurrent professor of the Department of Advanced Management of Cardiac Arrhythmias, the Department of Cardiac Remote Management System, and the Department of the Advanced Therapeutics for Cardiovascular Diseases, which is an endowment department supported by Boston Scientific Japan Co., Ltd. Dr. Tomita also received research grant from Abbott Medical Japan LLC. Other authors have no relevant disclosures.

## Supporting information


Figure S1

## Data Availability

The data supporting this study's findings are available on reasonable request from the corresponding author.
