# Supplementary figures and images for: Landiolol, an intravenous β1‐selective blocker, is useful for dissociating a fusion of atrial activation via accessory pathway and atrioventricular node
Source: J Arrhythm. 2023 Oct 3;39(6):937–46. doi: 10.1002/joa3.12934 (PMC10692861; doi:10.1002/joa3.12934)

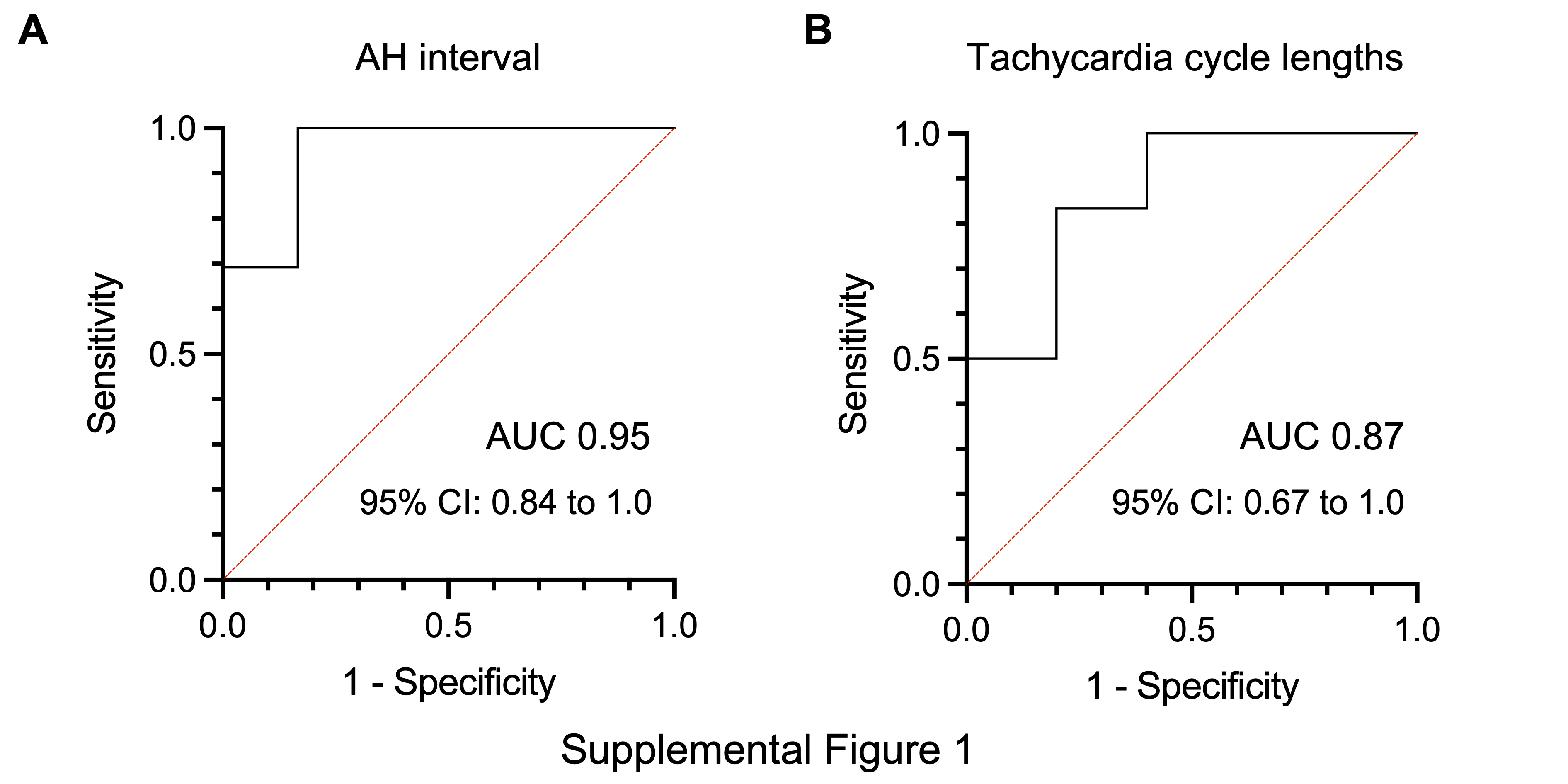

Supplement: Supplementary file 1 — Figure S1 [file JOA3-39-937-s001.tiff]
